# Developing a combined framework for priority setting in integrated health and social care systems

**DOI:** 10.1186/s12913-023-09866-x

**Published:** 2023-08-21

**Authors:** Marissa Collins, Micaela Mazzei, Rachel Baker, Alec Morton, Lucy Frith, Keith Syrett, Paul Leak, Cam Donaldson

**Affiliations:** 1 Yunus Centre for Social Business and Health, Glasgow Caledonian University, Glasgow, UK; 2 Department of Management Science, University of Strathclyde, Glasgow, UK; 3 Centre for Social Ethics & Policy, University of Manchester, Manchester, UK; 4 University of Bristol Law School, University of Bristol, Bristol, UK; 5 Directorate of Health and Social Care, Scottish Government, Edinburgh, UK

**Keywords:** Priority setting, Resource allocation, Integration, Economics, Decision science, Ethics, Law

## Abstract

**Background:**

There is an international move towards greater integration of health and social care to cope with the increasing demand on services.. In Scotland, legislation was passed in 2014 to integrate adult health and social care services resulting in the formation of 31 Health and Social Care Partnerships (HSCPs). Greater integration does not eliminate resource scarcity and the requirement to make (resource) allocation decisions to meet the needs of local populations. There are different perspectives on how to facilitate and improve priority setting in health and social care organisations with limited resources, but structured processes at the local level are still not widely implemented. This paper reports on work with new HSCPs in Scotland to develop a combined multi-disciplinary priority setting and resource allocation framework.

**Methods:**

To develop the combined framework, a scoping review of the literature was conducted to determine the key principles and approaches to priority setting from economics, decision-analysis, ethics and law, and attempts to combine such approaches. Co-production of the combined framework involved a multi-disciplinary workshop including local, and national-level stakeholders and academics to discuss and gather their views.

**Results:**

The key findings from the literature review and the stakeholder workshop were taken to produce a final combined framework for priority setting and resource allocation. This is underpinned by principles from economics (opportunity cost), decision science (good decisions), ethics (justice) and law (fair procedures). It outlines key stages in the priority setting process, including: framing the question, looking at current use of resources, defining options and criteria, evaluating options and criteria, and reviewing each stage. Each of these has further sub-stages and includes a focus on how the combined framework interacts with the consultation and involvement of patients, public and the wider staff.

**Conclusions:**

The integration agenda for health and social care is an opportunity to develop and implement a combined framework for setting priorities and allocating resources fairly to meet the needs of the population. A key aim of both integration and the combined framework is to facilitate the shifting of resources from acute services to the community.

**Supplementary Information:**

The online version contains supplementary material available at 10.1186/s12913-023-09866-x.

## Background

Integration of care services is a way of coping with the increasing demand on health and care services as people are living longer and, consequently, the number of people with long term, often, chronic conditions is rising [1]. In addition, it is a way of improving patient experience and outcomes by treating all the needs of a person together, instead of a more fragmented approach across different health and care systems [2].

Integration can be undertaken at different levels from micro to macro, and by using different structures of integration as shown by the following international examples. At the micro level, there is integration for the individual patient with care planning and personal health budgets, or self-directed care. At the meso level, there are disease specific models of integration spanning both community and health systems to improve outcomes for those with certain conditions, as used in the USA and Sweden [1]. At the macro level, there are wider population based models, for example, Nuka System of Care in Alaska which has multidisciplinary teams providing integrated health and care services; Counties Manukau in New Zealand which works with local providers to develop locality-based integrated health and care teams; [2] and, the Norrtaelje model in Sweden, where Stockholm County Council, responsible for healthcare services, and Norrtaelje Local Authority, responsible for social care services, formed a Joint Governing Committee for health and social provision for the population of Norrtaelje [3].

In the UK, how health and social care services have been integrated has varied. In Scotland, the setting for this paper, the move has been at a macro level where legislation was passed in 2014 to integrate adult health and social care services resulting in the formation of 31 new Integration Authorities (IAs) called Health and Social Care Partnerships (HSCPs), which became operational in 2016 [4]. A key aim of integration was to shift the balance of care from acute hospital services to community-based services within specific localities. 30 out of 31 HSCPs established an Integrated Joint Board (IJB), which is a separate legal entity and can act on its own behalf to make decisions about the functions and responsibilities of the HSCP. The legislation set out a minimum membership for the IJB, including representation from the Health Board and Local Authority and key stakeholders from the Third Sector, carers, and members of the public. In England, more recently, legislation was passed in April 2022 to establish Integrated Care Boards (ICBs) to replace Clinical Commissioning Groups (CCGs) and take on their commissioning functions. The ICBs are required to establish Integrated Care Partnerships (ICPs) which are statutory joint committees and bring together partners from across the system to develop an integrated care strategy [5].

Despite the move towards greater integration of health and social care organisations, one reality that cannot be escaped is that organisations have to make decisions around how to allocate scarce resources to meet the needs of their local population. The move towards greater integration does not eliminate resource scarcity and despite the usefulness of agencies, such as the National Institute for Health and Care Excellence (NICE),the Scottish Medicines Consortium (SMC) and the Scottish Intercollegiate Guidelines Network (SIGN) in making recommendations about adoption of programmes and services, decisions are still required at a local level to balance national recommendations against local needs and resources. HSCPs need to take account of a broader pathway of care, one that includes health and social care, which encompasses the wider wellbeing of the population, moving away from focussing on the contribution of medical interventions to health. Difficult choices need to be made about service provision and resource allocation that consider the range of different criteria applicable to both health and social care decision making.

There are different perspectives on how to facilitate and improve resource allocation and decision making in health and social care organisations in the context of limited resources, and structured processes for priority setting and resource allocation at the local level are still not widely implemented. Although a number of perspectives and frameworks have been used to inform priority setting, these will likely require development to be useful in new integrated health and social care contexts. Further, very few attempts to combine different frameworks exist in the literature and, when this has been done, the focus has been solely on healthcare settings.

This paper reports on the initial stages of work with new HSCPs in Scotland to develop a priority setting and resource allocation framework for use in these settings. We report on work undertaken to develop a combined framework for use in four HSCPs incorporating perspectives on priority setting from four disciplines: economics, decision science, ethics, and law, based on published literature. Our research team includes experts from each perspective. The implementation stage will form the basis of analysis in a subsequent paper.

The combined framework was developed with involvement from key stakeholders working with HSCPs at the local level, and from national organisations, including the Scottish Government and Healthcare Improvement Scotland. By bringing together the literature on priority setting and resource allocation and the views of key stakeholders, we developed a combined priority setting framework for use within local HSCPs.

### Setting

Integration of health and social care in Scotland was driven by the Public Bodies (Joint Working) (Scotland) Act 2014 [4] requiring 31 HSCPs to make joint decisions about integrated budgets for primary, community, social and some acute hospital care, managing approximately £9billion of resources annually. The remit for the Partnerships, working with at least a minimum set of delegated health and social care services, is to improve health and wellbeing outcomes for the local population, using locality planning to target resources accordingly. A key aim of the legislation was to facilitate shifts in the balance of care from acute settings to people’s own homes or similar community environments. Correspondingly, to aid such decision-making, and to accompany its integration legislation, the Scottish Government issued an Advice Note [6] outlining the key characteristics essential for a priority setting process. This recognised the need for processes to consider costs and outcomes, as well as the needs and values of local populations. This Advice Note states that key economic principles are to be incorporated in a priority setting process – opportunity cost and the margin. This process must facilitate a local review of existing services and resource allocation, addressing both investment and disinvestment decisions within the same process, and should also include the Partnership’s total resource (i.e., the resource attached to adult services within the control of the Partnership). In addition, it should consider an assets-based approach, a human rights-based approach, ethical considerations, and be practical and proportional to the type and scope of the decision being made. The Advice Note suggests a list of tools to consider for prioritisation including option appraisal, Programme Budgeting and Marginal Analysis (PBMA), Cost–Benefit Analysis, Social Return on Investment and Multi-Criteria Decision Analysis (MCDA) [6]. However, although the Advice Note outlined characteristics that highlight the importance and requirement for an explicit priority setting process, it stopped short of proposing an explicit framework for potential use at the local level for health and social care priority setting and resource allocation.

Notwithstanding this limitation, health and social care integration requires robust processes for allocating resources, as highlighted in the Advice Note, as difficult decisions need to be made about which services to fund and to what extent, and to identify which existing services to scale back. These changes were not accompanied with any increase in spending on health and social care in the short or medium term.

## Methods for developing the framework

The Advice Note, which was designed to facilitate the use of frameworks for priority setting by the Partnerships, formed the starting point of our scoping review of the academic literature. The Advice Note did not provide a comprehensive picture of frameworks for priority setting, thus the scoping review sought to provide further details and to fill any gaps. Two searches were conducted. Search one was on the specific tools listed in the Advice Note and search two was on the different perspectives that focus on priority setting (economics, decision science, ethics, and law) to capture any additional frameworks not mentioned in the Advice Note. Further information is provided in a supplementary information document outlining the search strategies.

**Fig. 1:**
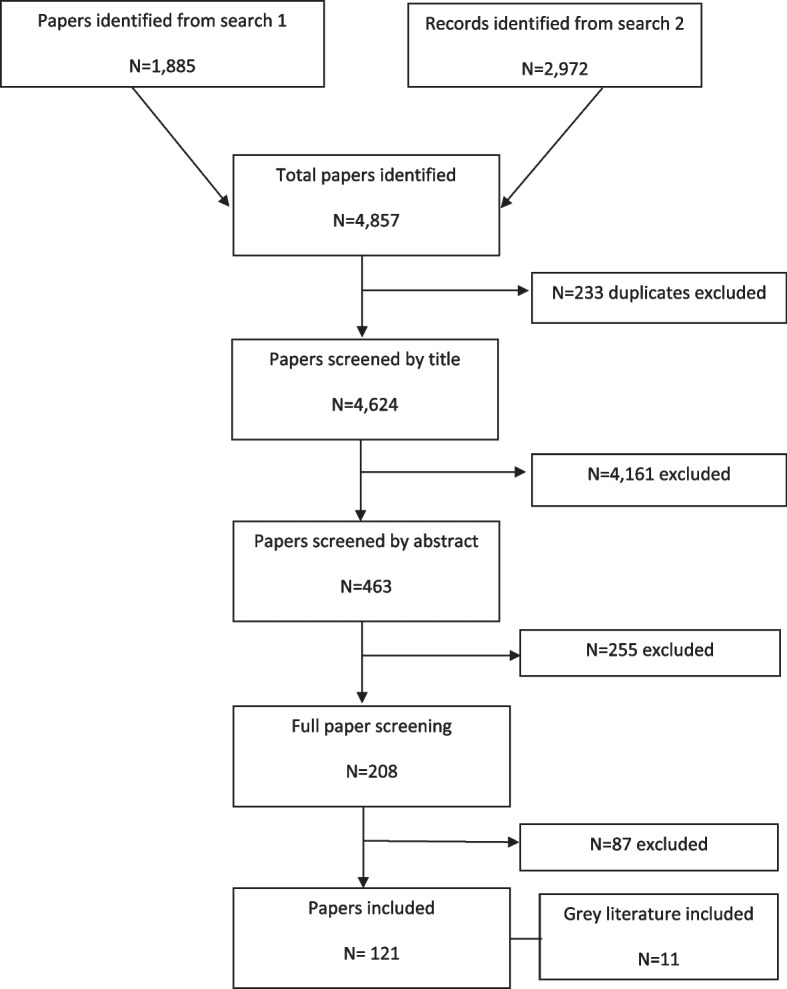
Findings from the scoping review

Key words and phrases from each discipline were used to search online academic databases, such as, MEDLINE, CINAHL and ProQuest. The searches were initially run for papers published up to July 2017 and the review has since been updated to identify papers published up to March 2022. Overall, a total of 121 full papers were included in the scoping literature review, as shown in Fig. 1. The papers were reviewed by MC with discussion with the project team around the literature identified and how it linked to the framework as this was developed. There was no quality assessment of the papers undertaken due to the wide scope of the literature searches and varying types of studies included in the review. The papers included were those considered to be relevant for priority setting and resource allocation at a local level. The reviews do not consider national level Health Technology Assessment (HTA) of healthcare interventions and drugs as these types of analysis (cost-utility analysis and cost-effectiveness analysis) are not conducted at a local level [7]. Papers were excluded for the following reasons: focus on research priorities, on HTA, on priority setting using QALYs, on life saving resources, on euthanasia, on end-of-life decision making, on organ transplantation, on pandemic and public health emergency decision making.

Alongside the scoping review, the development of the framework was started using an iterative process. The initial iterations began to incorporate principles, values and process stages found as literature was being reviewed. These were documented alongside examples and learning of where processes had been implemented. Table 1 outlines the timeline and steps in developing the framework.

**Table 1:** Timeline for developing the framework

July/August 2017	Literature Review underway
	Four iterations of the framework diagram drafted as literature is reviewed
September 2017	The fifth iteration of the diagram based on the literature presented to the project team
	A further diagram drafted after team discussion and prior to stakeholder workshop
	Stakeholder workshop (*N* = 18): expert presentations, small group exercise on what should be included in a priority setting framework, group discussion and presentation of draft framework
October 2017	Thematic analysis of the outputs from the stakeholder workshop
	Diagram developed based on the analysis of the stakeholder workshop
October/November 2017	Bringing it all together: Combining the diagram based on the literature review with the diagram from the stakeholder workshop
	Team discussion
	Final diagram developed prior to the implementation stage

Following team discussion, we identified a need to start the framework with underpinning principles, followed by the order of the stages. This discussion and input then informed the next draft of the framework where the underpinning principles were made more explicit and the structure of the framework was changed to improve the overall flow of the process.

A stakeholder workshop was then held with the aim of co-producing the framework.. Co-production in the context of research is about collaboration between different stakeholders and can be viewed as the “sharing of power”, with stakeholders and researchers working together [8] to combine the stakeholders’ knowledge gained through experience with more formal research knowledge [7]. The intention was that gathering and taking account of local and practical knowledge and views of key stakeholders would result in a framework that was more likely to be successfully implemented in practice.

It was intended that the framework would be implemented in four Partnership sites: Falkirk, North Lanarkshire, Western Isles and Clackmannanshire & Stirling. The Chief Officers in these areas had been approached prior to the development of the framework to discuss the project and their potential involvement in it. This was facilitated by our Scottish Government colleagues who had identified Partnerships that might need help in this area and would be willing to work with the project team.

To offer some degree of orientation as to purpose, a summary document of the approaches found in the literature was circulated to attendees in advance of the workshop, together with the Scottish Government Advice Note [6]. These materials were used as the basis for discussion on the day of the workshop.

At the beginning of the workshop, expert members of the project team presented a summary of each perspective related to their discipline—economics, decision science, ethics and law – and how it links to priority setting. This ensured that attendees were familiar with each perspective and facilitated discussion.

Attendees at the workshop included, colleagues from Scottish Government involved in health and social care integration, Healthcare Improvement Scotland (a national NHS organisation), academics, and health and social care professionals from the four HSCPs. They were placed in small groups made up of people from different areas and organisations. To avoid leading the participants but to facilitate discussion, the groups were asked to outline the stages involved in getting from “current allocation of resources” to “recommendations for change” on an otherwise blank diagram. This exercise was conducted to give participants the opportunity to build their own process and provide new insights. The groups were asked to discuss what should be included within such a priority setting process and to make notes on the diagram and feed back to the room. Finally, the draft framework developed from the literature review was presented by the project team to gather initial feedback on its content and design and to discuss where improvements could be made. The outputs from the workshop including the diagrams developed by each group were then typed up and thematically analysed to identify the main themes and how these could be incorporated into a single combined priority setting framework.

**Fig. 2:**
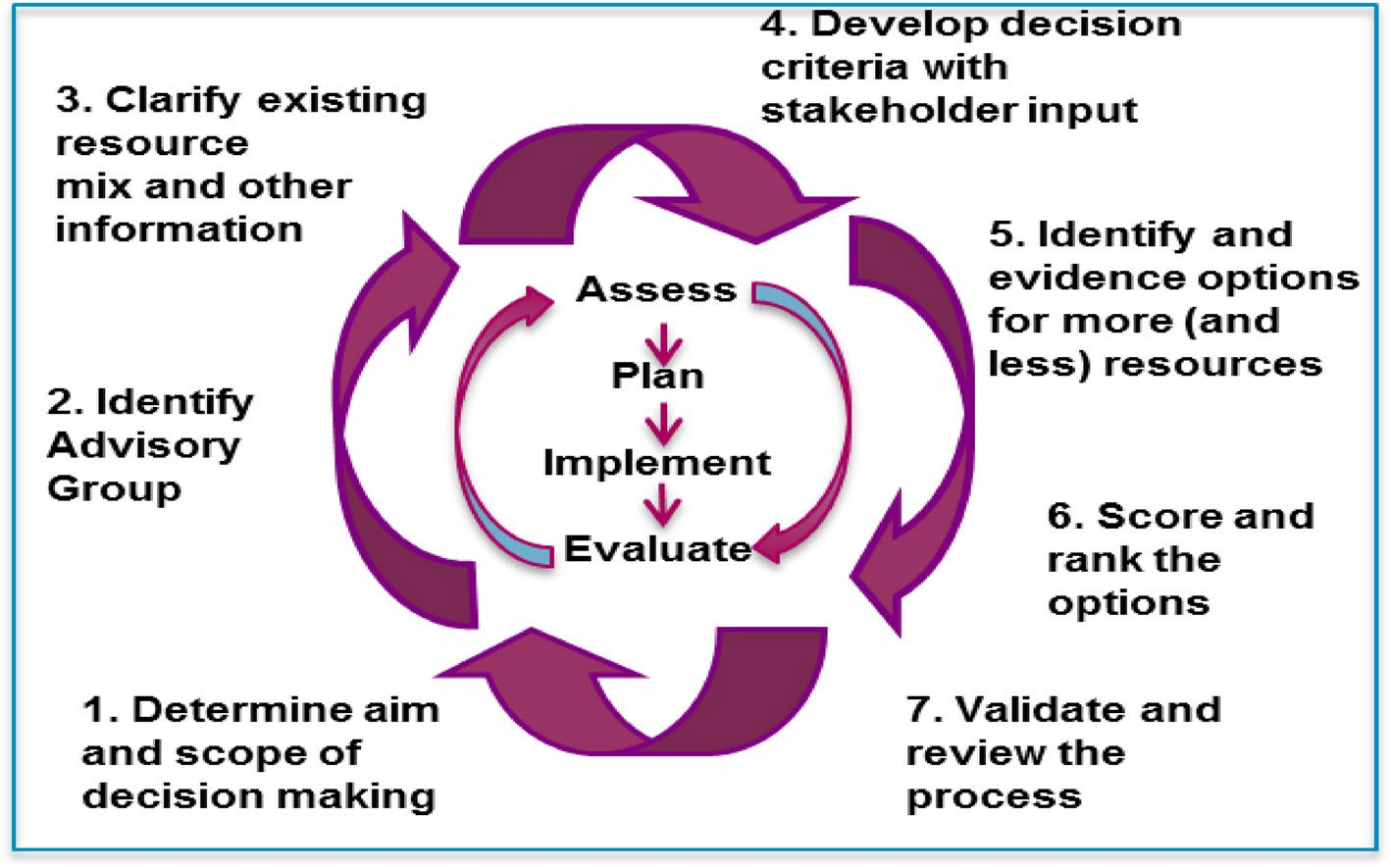
Project management of PBMA: the 7 steps (Adapted from [9])

The key findings from the literature review, discussions with the project team, and the diagrams and discussions from the stakeholder workshop were combined to produce a final framework for priority setting and resource allocation as shown in Fig. 2 below.

This paper will next outline the key findings from the literature review, the outputs from the stakeholder workshop, and the final framework.

## Findings from the literature

The key findings from the literature review are summarised, first, based on the principles from each perspective that are considered relevant for priority setting and were incorporated into the final framework. Secondly, an outline of the processes and key stages of priority setting from each perspective follows. These provided the foundations for the development of the draft framework and formed the basis for the summaries presented at the stakeholder workshop. There is overlap between each of the perspectives outlined below but, for ease of reporting, they have been separated out to highlight the specific processes, stages and principles that underpin each of the perspectives for developing the framework.

## Literature: aims and principles of priority setting

Economic approaches to priority setting are underpinned by two key principles – ‘opportunity cost’ and ‘the margin’ [10]. Opportunity cost refers to having to make choices on how resources are allocated within a fixed budget; certain opportunities will be taken up while others will not i.e. allocating resources in one way means that they are not being allocated in a different way [10]. The benefits, for example improvement in quality of life or in access to services, associated with opportunities that are not taken up are called opportunity costs. Thus, we need to know the costs and benefits from various health care activities. Once the costs and benefits are known, the assessment of costs and benefits ‘at the margin’ can be conducted. This marginal analysis focuses on the benefit gained from an incremental increase in resources, or benefit lost from an incremental reduction in resources [10]. This means that the application of economics, in this context, focuses upon the balance of health and social care services, not necessarily the introduction or elimination of a service in totality, fitting with the agenda of shifting the balance of care from acute settings to home and/or the community.

From decision science, the aim is to support decision makers in establishing preferences between options based on a set of objectives that have been identified. A key feature is the use of multiple criteria to make decisions and provide robust techniques for the weighting of criteria and scoring of options for change [11]. However, there is a need to understand other perspectives on priority setting to effectively defend investment and disinvestment decisions given that the goal of priority setting is not solely about maximising the benefits from the health services provided but may also be concerned with equity issues, such as looking to treat those in the greatest need, or to reduce health inequalities.

Moving away from the more technical approaches to priority setting, the main ethical value that is relevant for priority setting is justice [12]. The formal principle of justice states that cases which are the same should be treated alike and cases which are not the same can be treated differently [13]. This formal principle then needs to be specified and context specific theories of justice developed. Linking this to priority setting has shown that particular groups, such as the most severely ill, are generally prioritised by respondents in studies of societal values [14, 15]. The ethical issues raised are: what are like cases? And what specification of justice is appropriate in particular settings? Alongside these concerns, is the need to consider processes for priority setting, and the values that should underpin these processes.

We might also wish to take a human rights based approach, where we would look to achieve a fair distribution of resources [16]. Lie [17] considered how a human rights framework could be used to mobilise resources for health. A guiding principle in human rights is non-discrimination i.e., access to healthcare cannot be denied on the basis of race, religion, social and other status. Viewing ‘health as a human right’ does not enable particular decisions to be reached as to how scarce resources should be allocated among different patient groups, but it does ensure that rights, understood with reference to national and international standards, are put at the centre of policies and practice. In addition, a recent review of adult social care in Scotland was conducted, suggesting a further reform of adult social care, putting a human rights approach at the centre [18]. However, it is unclear at this stage how this links with priority setting and resource allocation and how it would be implemented in practice.

A further, overlapping, perspective is that of the law. Legal norms seek to ensure that decision-makers act in a procedurally just manner, for example by adhering to principles of transparency and participation. Priority-setting must also take place within relevant legal frameworks (such as those related to human rights or non-discrimination) and be based upon criteria which are relevant to the making of a priority-setting choice [19].

The aims and principles from each perspective provide a starting point for the development of the framework. The goal is to establish a fair process that will stand up to scrutiny from ethics and law and incorporate more technical aspects to achieve this from economics and decision science.

## Literature: process aspects of priority setting

We will now consider the priority setting process itself and the practical stages that need to be considered to give effect to the principles set out above.

From economics, PBMA attempts to connect economic principles (opportunity cost and the margin) with a project management framework to provide a structured process. PBMA starts by examining how resources are currently allocated and how potential changes in that mix could maximise the benefits from the services provided. PBMA asks five questions about resources, as shown in Table 2 [10].

**Table 2:** Using PBMA: five questions for localised priority setting process [10]

	PBMA addresses priorities from the perspective of resources:
1	What resources are available in total?
2	In what ways are these resources currently spent?
3	What are the main candidates for more resources and what would be their cost and effectiveness?
4	Are there any areas of care within the programme which could be provided to the same level of benefit but with fewer resources, so releasing those resources to fund candidates from (3)?
5	Are there any areas of care which, despite being effective, should have fewer resources because a proposal (or proposals) from 3 is (are) more effective for the resources spent?

Answering these questions is challenging and relies on careful project management. Therefore, alongside these questions there are seven steps of project management, as outlined in Fig. 2. These provide a generic structure for decision-makers to follow, with key activities for each step.

Substantial research has been undertaken on PBMA and priority setting in health [9, 20–24] however, it is not routinely used to set priorities within organisations [25]. The challenges of using PBMA to set priorities include: the need to create capacity within organisations for staff to complete the process alongside many other competing tasks, since lack of capacity can affect the membership of the Advisory Group and non-attendance at meetings, leading to the process stalling. Additionally, gathering data to inform the programme budget can be challenging, and will be even more so when social care is considered within the process, as the data is not as well established as that for health care.

A key advantage of using a structured PBMA process is that it can facilitate more transparent decision-making and allows explicit comparisons to be made among different options for investment and disinvestment.

From decision science, multi-criteria analysis (MCA) can be used to set out the problem, objectives, values, and options that decision makers are faced with in a clear and transparent way [26]. MCA is a qualitative process to describe the expected performance of options against criteria. MCDA provides similar stages to PBMA that can be undertaken to set priorities, outlined in Table 3, and includes the quantitative methods for scoring and weighting criteria and options. A focus for the development of the framework was to consider the quantitative aspects of scoring and weighting from MCDA to link it to the PBMA process and provide a full overview of potential approaches. The requirement to outline current resource use is not explicit in the MCDA process but it does include the use of criteria as a way of assessing benefits from different options and includes weighting of criteria and scoring of options.

**Table 3:** The main steps of MCDA (adapted from [27])

1	Defining the decision problem: clear description of the problem, and validate and report it
2	Selecting and structuring criteria: report and justify the methods to identify criteria and definitions
3	Measuring performance: report and justify the sources used to measure performance and the performance matrix
4	Scoring alternatives: report and justify the methods used for scoring
5	Weighting criteria: report and justify the methods used for weighting
6	Calculating aggregate scores: report and justify the aggregation function used
7	Dealing with uncertainty: report sources of uncertainty and the uncertainty analysis
8	Reporting and examining of findings: report and examine the MCDA method and findings

Another key distinction between MCDA and PBMA is the use of a performance matrix. The purpose of the matrix is to show the performance of each alternative against each criterion [27]. For example, Table 4 shows a performance matrix for five options for an elderly couple who are buying a new house. The housing options are compared in terms of the purchase price, convenience (i.e. availability of facilities), accessibility (i.e. on one level) and proximity to children in driving time (personal correspondence, Morton, A.). The purchase price and proximity to travel can be measured in natural units (monetary value and time). Accessibility is a discrete measurement (yes or no). Convenience is a constructed measurement e.g. assigning A for very convenient if at least three of the following facilities are within 1 km: supermarket, pharmacy, post office, clinic, and, if all four are within 2kms; assigning B if at least two of the above are within 1 km and at least three are within 2kms; assigning C if at least three of the above are within 3kms.

**Table 4:** Example of a performance matrix^a^

Housing option	Purchase price	Convenience	Accessibility	Proximity to children
1	£220,000	A	Yes	30 min
2	£180,000	B	Yes	30 min
3	£130,000	C	No	20 min
4	£120,000	C	No	40 min
5	£180,000	B	No	30 min

^a^Adapted from personal correspondence from Morton, A

In a performance matrix, the criteria are shown along the top of the table and the options in the first column. The information in the matrix can be converted to a numerical value. The main idea here is to construct scales (0–100) representing preferences for each option, with 0 being the worst performing level and 100 the best level. To facilitate this, it is useful to draw scales, ask individuals to score each option and then discuss the scores.

Weighting criteria is part of both MCDA and PBMA (to show the relative importance of each criterion). One criticism is that the techniques incorporated within a PBMA approach to weight criteria are not robust and could underestimate trade-offs between criteria, resulting in options that do not offer improvement in health, being scored high if they perform well in certain criteria [28]. One potential means to overcome this is to incorporate techniques from MCDA [11]. One such is swing weighting which is a systematic and theoretically well-grounded technique for weighting criteria. A swing is an increase in performance of an option against a criterion from the worst to the best performance level and a weight reflects the value of that swing, for example, we could look at the swing from worst to best price compared with a swing from worst to best convenience. If the swing on purchase price is preferred then this would be given a weight of 1 and convenience would then be assigned a fraction of that weight, for example, 0.85 (again decided by those involved in the decision-making); this would be repeated for each criterion. Once the scores and weights have been decided, the scores are multiplied by the weight attached to the criterion and summed across criteria [29].

There are tools and software that can help organisations use MCDA. One such tool, developed by the Health Foundation and the London School of Economics, is STAR – Socio-Technical Allocation of Resources [30]. This includes a freely available spreadsheet and guide for local organisations. However, the literature shows that expert involvement is required at the local level to assist with applying the framework and that this may not always be available and will require funding [29, 31]. Public Health England (PHE) launched a prioritisation framework to help local authorities make spending decisions across public health programmes. This is also based on MCDA and includes a spreadsheet and guidance on how to use the tool [32]. It is not clear if the tool has been used for priority setting at the local level.

PBMA and MCDA map out a process for priority setting and certain key activities to assess different options for investment and disinvestment within the same framework and these fit together well given the use of multiple criteria in both.

From the ethics literature, the approach which focusses on fairness and procedural justice is called Accountability for Reasonableness (A4R) which outlines procedural conditions that a priority setting process must meet to be considered fair, shown in Table 5 [33].

**Table 5:** Procedural conditions of Accountability for Reasonableness

Condition	Description
(a) Ensure publicity for the priority setting process	Make the priority setting process and decisions, and the rationales behind them, accessible to stakeholders and the local population
(b) Ensure relevance of the priority setting process	The priority setting process and resulting decisions should be based on principles, reasons, and evidence that fair-minded people agree are relevant
(c) Establish an appeals mechanism	The mechanism should allow people to challenge decisions within the prioritisation process and facilitate resolution of disputes, if necessary by revising decisions in light of further evidence
(d) Establish an enforcement mechanism	There is voluntary or public regulation of the prioritisation process and an appeals mechanism to ensure that the first three conditions are met

In addition to A4R, there have been attempts in the literature to map out social values that can be considered in priority setting processes. Clark and Weale [13] set out such a conceptual framework, including the process values (how decisions are made) of: transparency, accountability and participation. Similar to A4R, these values highlight that it is important for people to be aware of the reasons why decisions are made and so the process for decision-making is important to facilitate decisions being accepted by society., The conceptual framework also sets out content values of: clinical effectiveness, cost-effectiveness, justice/equity, solidarity and autonomy. The process and content values are often closely related and content values can usually be found in technical criteria used in a PBMA process. In practice, the ethical perspective has been more focussed on how decisions are made and whether the procedures used allow for fair decision-making processes.

The final perspective is legal scrutiny which can fulfil the enforcement condition of A4R and ensure the realisation of the other conditions of the model [19]. To this end, courts have provided an oversight role: where challenges to priority setting decisions are made, they will closely scrutinise both the fairness of the process used to make the decision and the application of the criteria by the organisation, although they usually remain reluctant (and lack the legal power) to challenge the outcome of the priority-setting process [34].

There have been suggestions in the literature to combine ethical evaluation with economic appraisal to facilitate the problems of shifting resources from one service to another by making the process fair and transparent and as a way of evaluating a PBMA process [35, 36]. There is also consideration of bringing MCDA and A4R together to meet the ethical demand for providing reasons why decisions were taken [37, 38].

Building on this, a comprehensive approach to priority setting is suggested, first by Peacock et al. [39] advocating for the inclusion of MCDA, A4R and Participatory Action Research (PAR) in to a PBMA process. Mitton [36] went further to consider PBMA, MCDA and A4R in developing a comprehensive approach to priority setting. This approach follows the basic steps of PBMA and highlighted that MCDA was already included within these stages and outlines how PBMA adheres to the conditions of A4R.

However, the literature on combined approaches tends to focus on evaluating whether a completed priority setting process meets the conditions of A4R. There have been arguments for bringing PBMA, MCDA and A4R together but no further frameworks have been developed explicitly for local level priority setting. In bringing these perspectives and accompanying principles and processes together into a combined framework, we argue this will encourage decisions to be made on the basis of a robust, fair and transparent process.

## Bringing it all together: the final framework

**Fig. 3:**
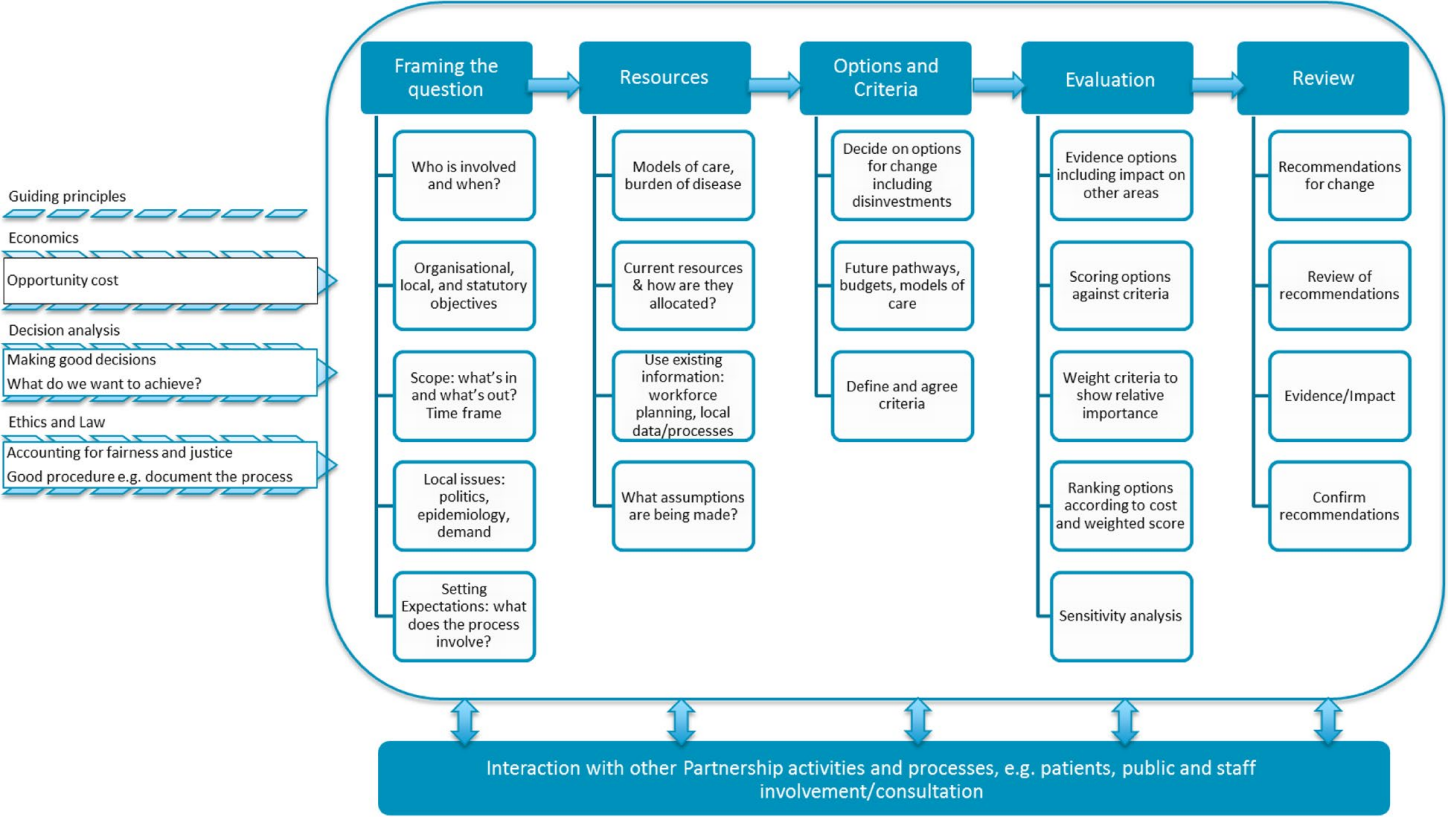
Combined framework for priority setting

The final combined framework, shown in Fig. 3, incorporates the findings from the literature review, discussions with the project team and the discussion and analysis of the outputs from the stakeholder workshop.

The framework starts with the guiding principles from the literature, taken from economics, decision science, ethics, and law. The key themes of “Framing the question”, “Resources”, and “Review”, were taken from an analysis of the outputs of the stakeholder workshop, incorporating what attendees considered to be important in a priority setting process to ensure that the process was practically valuable. The subsections under “Framing the question” and “Resources” were identified by the stakeholders as being important in setting up a priority setting process. These subsections allowed for the managing of expectations of those involved and assessing what is in and out of scope with regard to services and resources that are under the control of the Partnerships, as there are national priorities that must be resourced. The stakeholders considered how existing local information is considered within a process, for example, workforce planning; and what assumptions were being made around the resource use at this stage. Disinvestment was seen as being key to changing the mix of services and so was explicitly included in the framework under “Options and Criteria”. The stakeholders said that any changes made needed to take into account impact on the provision of other services, and this was included under “Evaluation”.

At the review stage, there is consideration and review of the recommendations from the process. There is no explicit appeals stage as the recommendations will then go to the IJB, the decision makers to determine whether they will proceed with the recommendations, and it is at this stage that any appeals will be brought forward. These meetings are public meetings, and all documentation is published online.

One key difference with the literature was that this indicated the inclusion of a set of criteria, such as, health gain or access to services, but this was not brought up by the stakeholders. Therefore, defining and agreeing criteria were included along with options for change. The need to evaluate the options for change by weighting criteria and scoring options was also not considered by the stakeholders, however, this is a key stage recommended in the literature and so an “Evaluation” stage was included to facilitate the ranking of options.

Throughout the process, appropriate consultation and involvement of staff and the public is essential to ensure adequate engagement and participation. This was considered in the literature and by the stakeholders. This was included along the bottom of the framework to show the need to consult at all stages and not just once final recommendations have been made. This is crucial to the process fitting the conditions of A4R and, thus, for the process to be considered fair. By including participation, it ensures that the framework considers the wider literature on values for priority setting [13].

To facilitate implementation of the framework, a guidance document was developed to provide further information for each stage of the process.. This included examples of criteria that could be used, a template business case, and templates for weighting the criteria and scoring the options.

## Discussion

Decision makers within health and social care settings are constrained by scarce resources, increased demand on services and the lack of systematic frameworks for the allocation of resources for commissioning that explicitly focus on trade-offs. While the integration of health and social care introduces complexities, it also represents a unique opportunity to think about and apply different perspectives and processes for priority setting. The legislation for the integration of health and social care services in Scotland sets a course through which to address the balance of care and to improve the use of increasingly scarce resources and outcomes for the population. Support was provided by the Scottish Government for the Partnerships, with points of contact and publication of Advice Notes to aid development of the appropriate structures and governance. The prioritisation Advice Note published by the Scottish Government points towards the implementation of more structured processes for priority setting at the local level but there is still a lack of explicit processes or frameworks for these new organisations to use. The combined framework for priority setting at a local level described in this paper seeks to fill this gap.

The stakeholders involved in the development of the framework were particularly receptive to working with us due to the changing policy and financial context. Decisions continue to be challenging as funding of health and social care organisations is increasingly squeezed, and disinvestment decisions are likely to increase. This participation allowed us to build on the four different perspectives from the literature – economics, decision science, ethics, and law – and approaches – PBMA, MCDA and A4R to develop the combined framework shown in Fig. 3. This element of co-production with those working in the Partnerships provided an insight into their perceptions of priority setting processes and what they think is important and would like to see included in such a framework. Our combined framework offers an in-depth and practical process for setting priorities and allocating resources. It incorporates a number of additional stages (both from the literature and from the stakeholders involved in the development) compared to PBMA and MCDA approaches to ensure the conditions of A4R are met. Therefore, it has built on these existing processes to include further detail within the same diagram for users to consider, while also being explicit about the principles which underpin the process. By including these at the start, users can have them in mind as the process is worked through. With the integration of health and social care services, a combined and explicit framework may facilitate the coming together of different organisations involved in the HSCPs to agree the process by which to make resource allocation decisions.

Our approach is a novel one. The development of STAR, as referred to in the literature section, is an attempt to introduce MCDA to local organisations. However, the literature shows that local organisations require external support for the use of this process [26]. Our aim was to produce a framework that local organisations can adopt without or with limited external input, hence the use of co-production in the development stage. There have been attempts to combine PBMA with A4R [22, 35–40]. However, these have generally used PBMA to set priorities and then evaluated the process using A4R, therefore using A4R as a checklist to ensure ethical priority setting rather than combining the principles and conditions within the same process. Others have considered MCDA with A4R [33] but, again, to evaluate whether the process had met the A4R conditions. Therefore, although Youngkong et al. do consider a range of disciplines, they do not explicitly bring them together. The aim of our framework is to provide a single combined and co-produced framework for those at the local level to use and to ensure that the relevant principles and stages are explicitly considered at the start of any priority setting and resource allocation process, are embedded with one another, and thus, fully considered throughout.

There are, of course, criticisms of the processes and principles involved in priority setting from each perspective, that we have not outlined fully in this paper. These criticisms are usually outlined for each separate perspective and, as we can see from the literature, when they have been considered together it is not as one explicit process. In bringing them together, the ideal is that we can overcome these criticisms to improve priority setting and resource allocation at a local level.

The involvement of local level professionals should ensure that this framework is appropriate for use in the context of integrated health and social care services, but further testing with representatives from the full range of stakeholders involved in IJBs will be required. The outstanding point is how much concurrence there is between a framework emerging from a bottom-up process devised by stakeholders and what has emerged in the published literature in what might be characterised as the view of ‘experts’. However, one key finding was that the stakeholders did not consider using criteria as part of the priority setting process and did not consider the evaluation of options for change. This highlights the importance of bringing together stakeholders and experts, as these are key stages set out in the literature (on PBMA, MCDA and A4R) and also in the Advice Note, but which were not highlighted at the stakeholder workshop. Although this may suggest a discord between expert guidance and those working at the local level, it has been shown that working without criteria can be problematic [37, 38].

The next step of implementing the combined framework to allow for a real world test with the four HSCPs involved in developing it, will be analysed and written up for a subsequent journal article. This involves working through the different stages of the process in an area of service provision chosen by those working at the local level. This could show whether a more robust process for priority setting and resource allocation facilitates a shift in how scarce resources are used and what changes to the provision of services take place. Documenting the process in each area will allow us to determine how the framework will be practically implemented and the barriers and facilitators to doing so. In addition, the combined framework could incorporate the views and values of those involved in decision making at a local level and those affected by decisions.

## Conclusion

The integration agenda for health and social care is an opportunity to develop and implement a combined and systematic framework based on several disciplines and approaches for the common purpose of sustaining publicly-funded services whilst ensuring they meet the needs of the population fairly. The combined and co-produced framework could facilitate the shifting of resources from acute to community services, a key aim of integrating health and social care services. Future work on the implementation of the framework at the local level is now required to determine how it would work in practice.

### Supplementary Information

**Additional file 1:** **Supplementary Information.** Scoping Review.

## Data Availability

Not applicable.

## Abbreviations

HSCPs: Health and Social Care Partnerships
ICBs: Integrated Care Boards
CCGs: Clinical Commissioning Groups
ICPs: Integrated Care Partnerships
NICE: National Institute for Health and Care Excellence
SMC: Scottish Medicines Consortium
PBMA: Programme Budgeting and Marginal Analysis
MCA: Multi-Criteria Analysis
MCDA: Multi-Criteria Decision Analysis
STAR: Socio-Technical Allocation of Resources
PHE: Public Health England
A4R: Accountability for Reasonableness
