# Supplementary material for: Developing a combined framework for priority setting in integrated health and social care systems
Source: BMC Health Serv Res. 2023 Aug 21;23:879. doi: 10.1186/s12913-023-09866-x (PMC10440867; doi:10.1186/s12913-023-09866-x)
Supplement: Supplementary file 1 — Additional file 1: Supplementary Information. Scoping Review. [file 12913_2023_9866_MOESM1_ESM.docx]

**Supplementary Information: Scoping Review**

**Inclusion and exclusion criteria:**

Include papers:

- With a focus on priority setting at the local level in the public sector
- which account for scarcity of resources
- from any country i.e., not just limited to the UK
- quantitative and qualitative approaches to priority setting

Exclude papers:

- Not in the English language
- Discussion and opinion papers

**Search 1: Specific tools**

| Keywords |  |  |
| --- | --- | --- |
| 1. Economic |  | Program* Budget* Marginal Analysis OR PBMA OR Social Return Investment OR SROI OR cost benefit analysis |
|  |  |  |
| 1. Decision analysis |  | Multi criteria decision analysis OR MCDA |
|  |  |  |
| 1. Ethics |  | Accountability Reasonableness OR human right* OR asset* based |
|  |  |  |
| 1. Priority setting |  | Priority OR priorities OR priority setting OR decision making OR decision* OR resource allocation |
|  |  |  |
| 1. Resources |  | Scarcity OR scarce OR limit* OR scarce resource* OR limited resource |
|  |  |  |
| 1. Setting |  | Public sector OR health care OR social OR social care OR social work OR community |

1 Or 2 OR 3 AND 4 AND 5 AND 6

**Search 2: Perspectives**

| Keywords |  |  |
| --- | --- | --- |
| 1. Economic |  | Economic* |
|  |  |  |
| 1. Decision analysis |  | Decision analysis OR decision analytic* |
|  |  |  |
| 1. Ethics |  | Ethics OR ethical OR ethic* OR moral* |
|  |  |  |
| 1. Law |  | Law OR legal OR judicial |
|  |  |  |
| 1. Priority setting |  | Priority OR priorities OR priority setting OR decision making OR decision* OR resource allocation |
|  |  |  |
| 1. Resources |  | Scarcity OR scarce OR limit* OR scarce resource* OR limited resource |
|  |  |  |
| 1. Setting |  | Public sector OR health care OR social OR social care OR social work OR community |

1 Or 2 OR 3 OR 4 AND 5 AND 6 AND 7
